# Risk factors and predictive model for pulmonary arterial hypertension in adult idiopathic-inflammatory-myopathy patients: A cross-sectional study

**DOI:** 10.1016/j.clinsp.2025.100621

**Published:** 2025-03-25

**Authors:** Junyu Liang, Xiaoqun Ba, Liyan Wan, Xiao Cui, Ye He, Lanlan Xiao, Yini Ke, Hanyin Zhang, Heng Cao, Jin Lin

**Affiliations:** a Department of Rheumatology, The First Affiliated Hospital, Zhejiang University School of Medicine, Hangzhou, Zhejiang Province, , PR China; b Department of Pathology, the First Affiliated Hospital, Zhejiang University School of Medicine, Hangzhou, Zhejiang Province, PR China; c Department of Cardiology, the First Affiliated Hospital, Zhejiang University School of Medicine, Hangzhou, Zhejiang Province, PR China

**Keywords:** Idiopathic inflammatory myopathy, Pulmonary arterial hypertension, Interleukin-17, Anti-SRP antibody, Aging

## Abstract

**Objective:**

To identify clinical and laboratory risk factors for Pulmonary Arterial Hypertension (PAH) in Idiopathic-Inflammatory-Myopathy (IIM) patients as well as construct a predicting model for PAH.

**Methods:**

An IIM cohort in southeastern China was established, including 504 adult IIM patients who met the inclusion and exclusion criteria, and were hospitalized at four divisions of the First Affiliated Hospital, Zhejiang University School of Medicine (FAHZJU) from January 1st, 2018, to April 30st 2022. Serum cytokine profiles, myositis-specific antibodies as well as other factors of patients who met the inclusion and exclusion criteria were collected and analyzed.

**Results:**

Of the 504 adult IIM patients, 25 IIM patients developed PAH and 48.0 % of them deceased within six months. IIM patients complicated with PAH were found to suffer from worse outcome (*p* < 0.001). After multivariate logistic regression analysis, age (*p* < 0.001), bacterial infection (*p* = 0.005), MYOACT score (*p* = 0.009), Interleukin (IL)-17A (*p* = 0.017), anti-SRP antibody (*p* = 0.011) and steroid monotherapy (*p* = 0.001) were identified as factors significantly associated with PAH in IIM patients. A “BAIMS” model was constructed by including the above six items to predict PAH with a cut-off value of ≥ 3 and an Area Under the Curve (AUC) of 0.877.

**Conclusion:**

PAH is a rare but potentially fatal complication in IIM patients. Aging, complication of bacterial infection, elevated disease activity, increased serum IL-17A level, positivity of anti-SRP antibody and steroid monotherapy were significantly correlated with development of PAH in IIM patients. In addition, the BAIMS model was found valuable in prediction and early-identification of PAH in IIM patients.

## Highlights

• Systemically identify risk factors for PAH among IIM patients in southern China.
• Anti-SRP antibody and Interleukin (IL)−17A are correlated with IIM-related PAH.
• The BAIMS model is valuable in prediction and early-identification of PAH in IIM.

## Introduction

Pulmonary Arterial Hypertension (PAH) is a rare dyspnea-fatigue syndrome featuring pathological remodeling and increased pulmonary vascular resistance in the small pulmonary arteries, which frequently leads to increased right ventricular afterload, subsequent right-sided heart failure and death if not timely and effectively treated.^1^, ^2^, ^3^ The pathophysiological mechanism behind PAH is rather complex and to some extent unclear. Endothelial dysfunction, smooth muscle cell proliferation, fibroblast activation, chemokine and cytokine signaling, ion channel activity as well as multiple epigenetic alterations all contributed to the development of PAH.^4^ Connective Tissue Disease (CTD) has also been recognized an important contributor to PAH in addition to the idiopathic PAH, PAH secondary to drugs, infection, left heart disease, lung disease, hypoxia, obstruction of pulmonary artery.^5^ The complicated mechanism and the heterogeneous subtypes of PAH made the treatment difficult and unsatisfying in many cases.

In clinical practice, Systemic sclerosis (Ssc) was found to be more frequently complicated with PAH among various CTDs.^6^ Resembling the heterogeneity of PAH, Idiopathic Inflammatory Myopathy (IIM) is also a group of autoimmune abnormalities characterized by muscular and/or cutaneous involvements, including multiple subtypes like Dermatomyositis (DM), Amyopathic Dermatomyositis (ADM), Polymyositis (PM), Inclusion Body Myositis (IBM) as well as Immune-related Necrotizing Myopathy (IMNM).^7^ Extramuscular and extracutaneous involvements are also commonly seen in IIM patients, with Interstitial Lung Disease (ILD) and its Rapid Progression (RP-ILD) being research focus.^8^ However, several IIM patients complicated with PAH were also identified in clinical practice, a large proportion of them failed to receive timely PAH-targeted therapy and deceased within short period of time. Meanwhile the understanding in pathophysiological mechanisms, biomarkers, medications, prognostic factors, predictive models of PAH in IIM patients remained unknown. In this context, a large IIM cohort, which could reflect the IIM spectrum in southeastern China, was established to probe into risk factors for PAH in a comprehensive manner and construct a predictive model for PAH in IIM patients.

## Patients and methods

### Patients

To establish an adult IIM cohort, the authors acquired medical records and follow-up documents of adult patients who were hospitalized at Yuhang, Qingchun, Chengzhan and Zhijiang divisions of FAHZJU with discharge diagnosis of Dermatomyositis (DM), Amyopathic Dermatomyositis (ADM), Polymyositis (PM), Inclusion Body Myositis (IBM) as well as Immune-Mediated Necrotizing Myopathy (IMNM) from January 1st 2018 to April 30st 2022. The patients in FAHZJU were mostly from Zhejiang province and the adjacent Anhui, Henan, Fujian, Jiangxi and Jiangsu provinces, which reflect, to some extent, the well-beings and disease spectrum of southeastern China. The inclusion criteria were as follows: 1) Age over 18-years-old; 2) The definite/probable diagnosis of DM, ADM, PM, IBM or IMNM satisfying the 2017 ACR/EULAR classification criteria, as validated by two experienced rheumatologists (Heng Cao and Yiduo Sun)^9^ ; Meanwhile exclusion criteria encompassed: 1) Clarified overlap syndromes with other Connective Tissue Diseases (CTDs); 2) Secondary myopathy owing to thyroid dysfunction, strenuous exercise, drug-induced myositis (i.e., Chinese herbal medicine, lamivudine), inherited metabolic disorders, etc.; 3) Hospitalization for reasons unrelated to myositis and its complications such as fracture, pregnancy, cataract and acquired immunodeficiency syndrome, due to insufficient medical records for this study; 4) Loss to follow-up without death from any cause within three months after hospital admission. The research protocol was approved by the Institutional Review Board (IRB) of FAHZJU (Reference Number: 2022‒237) and conformed with the Declaration of Helsinki as well as the STROBE Statement. Written informed consent on utilizing and publishing clinical and laboratory data was obtained from all the included patients at hospitalization.

### Clinical assessments

Clinical records of all the enrolled patients were screened and assembled through the Electronic Medical Record (EMR) system of the four divisions of FAHZJU. Data including demographic information, complications, disease activity assessment, laboratory or radiological findings as well as immunosuppressive medications were extracted from the EMR system and afterwards analyzed. Survival data were obtained from the follow-up records in multiple divisions. The detailed process of follow-up has been documented in preceding study.^10^ The end of follow-up was defined as death from any cause, loss to follow-up, or closure of follow-up for the purpose of this study (October 31st, 2022).

Baseline disease activity assessment, recording of muscular and cutaneous manifestations, laboratory and radiological detections, etc. were finished within the first week after hospital admission. Baseline IIM disease activity was routinely measured using the Myositis Disease Activity Assessment Visual Analogue Scales (MYOACT). The MYOACT score was assessed by drawing vertical marks on a series of 10-cm lines, including constitutional, musculoskeletal, cutaneous, gastrointestinal, pulmonary, and cardiac. This comprehensive assessment allows physicians to acquire an overall severity of the ongoing disease activity.^10^ Identification of Myocardial Involvement (MI) and PAH was made by a cardiologist (Xiao Cui). To be exact, identification of PAH was mostly based on the estimation of systolic Pulmonary Arterial Pressure (sPAP) via echocardiography, which was fulfilled by a qualified sonographer as described in the 2015 ESC/ERS guideline.^11^ Most of the PAH patients (mild and moderate PAH patients in particular) was afterwards validated by the Right Heart Catheterization (RHC).^12^ Patients were thus stratified by sPAP into three groups, mild (30–50 mmHg), moderate (51–70 mmHg), severe (> 70 mmHg).^11^ In addition, patients with idiopathic PAH, congenital heart disease and left ventricular systolic dysfunction were not included into the IIM-PAH group. Meanwhile identification of MI was based on criteria listed in preceding research.^13^ The adult IIM cohort was thus divided into PAH group and non-PAH group (control group). Interstitial Lung Disease (ILD) and its rapid progression (RP-ILD) were screened and confirmed by experienced respiratory specialists (Yake Yao ang Bingjue Ye) using lung High-Resolution Computed Tomography (HRCT), the detailed protocol was presented in preceding studies.^10^^,^^14^ Diagnosis of bacterial, fungal, or tuberculosis infection was comprehensive decision according to microbiological findings in sputum, urine or blood, typical clinical manifestations, radiographic as well as laboratory alterations. Epstein-Barr Virus (EBV) and Cytomegalovirus (CMV) infection was identified based on the detection of serum antibody and DNA. Each patient in this cohort received immunosuppressive medications as described in Supplementary Table 1.

### Laboratory detections

To acquire peripheral lymphocyte subsets, cytokines as well as profiles of Myositis-Specific Antibodies (MSAs) and Myositis-Associated antibodies (MAAs) in this cohort, serum samples were drawn and detected within the first week after hospital admission. Detailed protocols of the regarding experiment could be seen in Supplementary Data 1.

### Statistical analysis

Statistical analysis was carried out utilizing SPSS 22.0 (Chicago, IL, USA), Graphpad Prism 9.0 and R 3.6.1. In comparison between PAH and non-PAH patients, independent sample *t*-test, Mann-Whitney U test as well as Chi-Square test and Fisher's exact test were used as per the characteristics of data. The univariate and the following multivariate logistic regression analyses were used for identifying significant factors correlated with development of PAH in IIM patients. Explanatory factors with *p* < 0.05 in comparisons or univariate analyses were entered into the multivariate analysis for further verification of PAH-related factors in IIM. Receiver Operating Characteristic (ROC) curve analysis was used to quantify the predictive value of continuous variables. Meanwhile decision tree analysis (using the Chi-Squared Automatic Interaction Detection approach to grow the tree) and ROC curve analysis was applied to double-check the cutoff value and predictive efficacy of the model for prediction and early identification of PAH. Survivals among different subgroups were evaluated by the Kaplan-Meier method with log-rank test. All tests were two-sided, and *p* < 0.05 was deemed statistically significant.

## Results

### Patient features

**Fig. 1:**
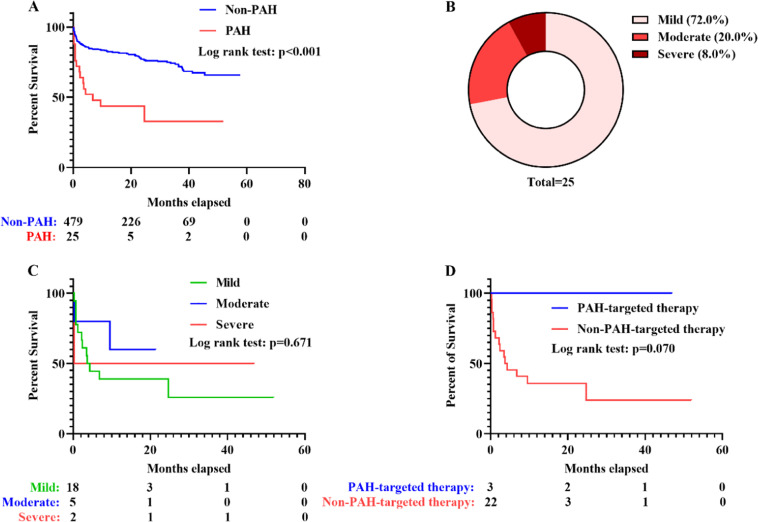
**Survival of IIM patients of different subgroups.** (A) Survival of IIM patients with or without PAH. (B) Distribution of mild, moderate and severe PAH in IIM patients with PAH. (C) Survival of IIM patients with mild, moderate or severe PAH. (D) Survival of IIM patients with PAH receiving or not receiving PAH-targeted therapy. IIM, Idiopathic Inflammatory Myopathy; PAH, Pulmonary Arterial Hypertension.

After checking with the inclusion and exclusion criteria, this study finally encompassed 504 adult IIM patients (Supplementary Fig. 1), including 308 with DM, 99 with PM, 58 with ADM, 37 with IMNM and two with IBM. Three hundred and forty-three (68.1 %) were females and the median age of all the included patients was 57.00 (49.00, 66.00) years old. In follow-up, 127 patients (25.2 %) deceased, and the medium follow-up time was up to 18.15 (7.91, 31.93) months. Within the 504 patients, 25 patients (5.0 %) developed PAH, and the 479 patients without PAH constituted the control group. Identification of PAH, in particular, was made after collection of serum samples, disease activity assessment, lung HRCT, etc. IIM patients complicated with PAH were found to suffer from more unfavorable outcome (*p* < 0.001, Fig. 1A), with 48.0 % of PAH patients deceased within six months. As per the grading criteria listed above, two patients suffered from severe PAH, five were subcategorized as moderate PAH, 18 were mild PAH (Fig. 1B). Among all these IIM patients suffering from PAH, patients with mild and moderate PAH received RHC for further verification. Two patients with severe PAH refused to receive RHC owing to the severity of disease and unsatisfying overall status. However, no statistically significant difference was identified among survivals of these three subgroups (*p* = 0.671, Fig. 1C). The percentage of PAH patients using PAH-targeted therapy was quite limited, only three patients (12.0 %), including one severe PAH patients using Macitentan and two moderate PAH patients using Ambrisentan, received timely PAH-targeted therapy. All the three of them lived over 16-months in follow-up. Owing to the small sample size, nevertheless, no significant difference was found between survival of patients receiving PAH-targeted therapy and that of patients without PAH-targeted therapy (*p* = 0.070, Fig. 1D).

### Clinical factors correlated with PAH in IIM patients

**Fig. 2:**
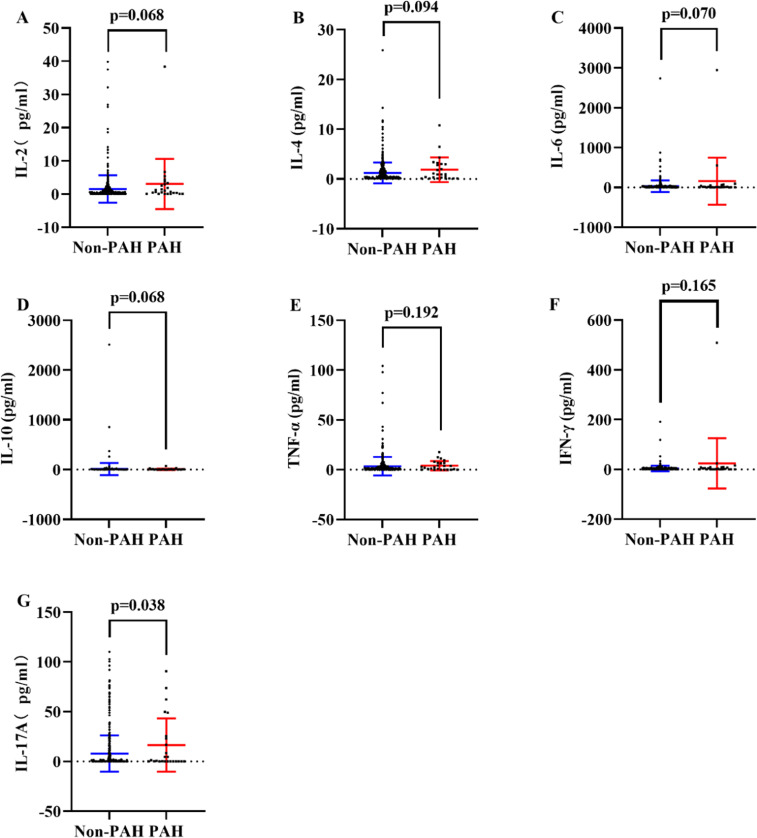
**Comparisons of serum cytokines in IIM patients with or without PAH.** (A) Comparison of serum IL-2 between PAH and non-PAH groups. (B) Comparison of serum IL-4 between PAH and non- PAH groups. (C) Comparison of serum IL-6 between PAH and non- PAH groups. (D) Comparison of serum IL-10 between PAH and non- PAH groups. (E) Comparison of serum TNF-α between PAH and non-PAH groups. (F) Comparison of serum IFN-γ between PAH and non- PAH groups. (G) Comparison of serum IL-17A between PAH and non-PAH groups. IIM, Idiopathic Inflammatory Myopathy; PAH, Pulmonary Arterial Hypertension; IL, Interleukin; TNF, Tumor Necrosis Factor; IFN, Interferon.

To acquire an initial understanding on clinical manifestations as well as imaging and laboratory alterations associated with PAH in IIM patients, a comparison between PAH and non-PAH patients was implemented and revealed that patients who developed PAH were comparably older (*p* < 0.001), had less joint pain (*p* = 0.023), more complications of bacterial infection (*p* < 0.001), RP-ILD (*p* = 0.003), MI (*p* < 0.001) and gastrointestinal hemorrhage (*p* = 0.029), elevated MYOACT score (*p* < 0.001), higher proportion of peripheral CD3^+^CD8^+^ lymphocytes (*p* = 0.012), lower CD4^+^/CD8^+^Ratio (*p* = 0.043), lower proportion of CD3^-^CD19^+^ lymphocytes (*p* = 0.001), higher serum levels of Interleukin (IL)−17A (*p* = 0.038, Fig. 2G), C-reactive protein (CRP, *p* = 0.004), CA125 (*p* < 0.001) and creatine kinase (CK, *p* = 0.024), more positivity of anti-SRP antibody (*p* = 0.034), more usage of steroid monotherapy (*p* = 0.001) as well as less usage of steroid + Disease Modifying Anti-Rheumatic Drugs (DMARDs, *p* = 0.001) (Supplementary Table 2).

The univariate logistic regression analyses for PAH indicated that age (*p* < 0.001), bacterial infection (*p* < 0.001), EBV infection (*p* = 0.043), RP-ILD (*p* = 0.004), MI (*p* < 0.001), gastrointestinal hemorrhage (*p* = 0.016), MYOACT score (*p* < 0.001), peripheral CD3^+^CD8^+^lymphocytes (*p* = 0.033), peripheral CD3^-^CD19^+^ lymphocytes (*p* = 0.003), IL-6 (*p* = 0.023), IL-17A (*p* = 0.033), CRP (*p* = 0.001), Erythrocyte Sedimentation Rate (ESR, *p* = 0.025), ferritin (*p* = 0.020), anti-SRP antibody (*p* = 0.019), usage of steroid monotherapy (*p* = 0.001) and steroid + DMARDs (*p* = 0.003) were correlated with development of PAH in IIM patients (Supplementary Table 3). The subsequent multivariate logistic regression analysis identified age (*p* < 0.001), bacterial infection (*p* = 0.005), MYOACT score (*p* = 0.009), IL-17A (*p* = 0.017), anti-SRP antibody (*p* = 0.011) and steroid monotherapy (*p* = 0.001) as clinical or laboratory factors that were significantly associated with development of PAH in IIM patients (Table 1).

**Table 1:** Multivariate logistic regression analysis of PAH among IIM patients.

Factors	p-value	OR value	95 % Confidence Interval
Age (y)	<0.001	1.084	1.037∼1.132
Bacterial infection	0.005	4.225	1.558∼11.459
MYOACT score	0.009	1.165	1.039∼1.305
IL17A (pg/mL)	0.017	1.023	1.004∼1.042
SRP	0.011	6.556	1.546∼27.802
Steroid monotherapy	0.001	4.919	1.845∼13.115

*PAH, Pulmonary Artery Hypertension; IIM, Idiopathic Inflammatory Myopathy; OR, Odds Ratio; y, years; MYOACT, Myositis Disease Activity Assessment Visual Analogue Scales; IL, Interleukin.

### Predictive model for PAH in IIM patients

**Fig. 3:**
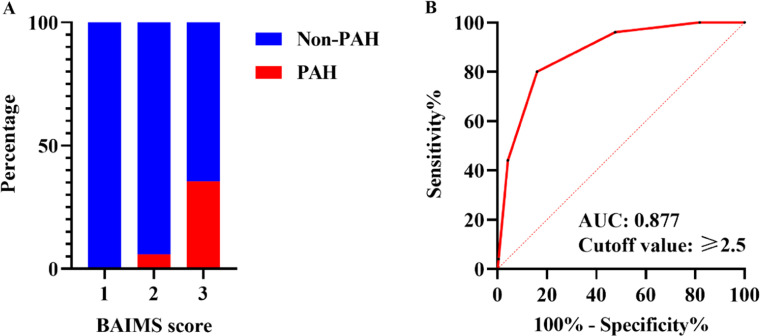
**Evaluation of BAIMS model in predicting PAH in IIM patients.** (A) Distribution of PAH and non-PAH patients in different BAIMS score groups. (B) ROC curve of BAIMS model predicting PAH. PAH, Pulmonary Arterial Hypertension; IIM, Idiopathic Inflammatory Myopathy; ROC, Receiver Operating Characteristic; AUC, Area Under the Curve.

**Fig. 4:**
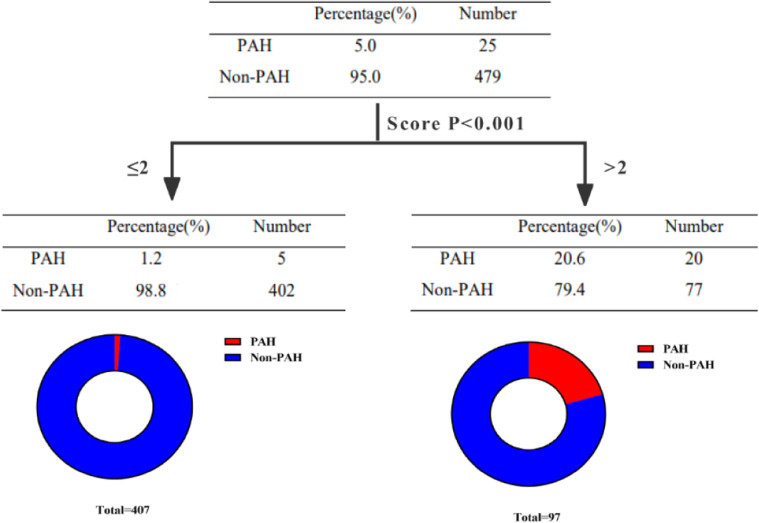
**Decision tree analysis for evaluation of BAIMS model in IIM patients.** IIM, Idiopathic Inflammatory Myopathy; PAH, Pulmonary Arterial Hypertension.

Utilizing ROC curve analysis, the optimal cut-off value of age for PAH was ≥ 66.5, with a sensitivity of 68.0 % and a specificity of 79.7 %. The Area Under the Curve (AUC) was 0.775 (Supplementary Fig. 2A). The optimal cut-off value of MYOACT score for PAH was ≥ 8.5, with a sensitivity of 84.0 %, a specificity of 53.0 %, and an AUC of 0.729 (Supplementary Fig. 2B). Meanwhile the optimal cut-off value of IL-17A for PAH was ≥ 0.14, with a sensitivity of 60.0 %, a specificity of 60.5 %, and an AUC of 0.609 (Supplementary Fig. 2C). To develop a practical scoring system for prediction of PAH, the authors rounded up the cutoff values of age, MYOACT score and IL-17A to > 65, > 8 and > 0.15, respectively. Each were allotted one point. Identification of bacterial infection, positivity of anti-SRP antibody and steroid monotherapy were as well allotted one point, respectively. Each included patient was then attributed a cumulative score with maximum score of six and minimum score of zero. The scoring system was named “BAIMS” arising from the initials of bacterial infection, age, IL-17A, MYOACT score, SRP and steroid monotherapy (Supplementary Table 4). Afterwards, the predictive value of BAIMS model was evaluated in the 504 patients. Only 0.4 % of the 252 patients with BAIMS score of 0∼1 developed PAH. In contrast, 35.5 % of the 31 patients with maximum range of BAIMS score (4∼5) were found to suffer from PAH (Supplementary Table 5, Fig. 3A). The decision tree analysis similarly revealed a cutoff value of > 2 (equal to ≥ 3 in this case) and identified that only 1.2 % of IIM patients with BAIMS score ≤ 2 developed PAH. Meanwhile IIM patients with BAIMS score >2 showed much higher tendency for PAH (20.6 %) (Fig. 4). For further validation, the following ROC curve analysis (Fig. 3B) as well revealed a cutoff value of ≥ 2.5 (equal to ≥ 3), AUC of 0.877, sensitivity of 80.0 %, specificity of 83.9 %, Positive Predictive Value (PPV) of 20.6 %, Negative Predictive Value (NPV) of 98.8 % and accuracy of 83.7 %.

## Discussion

PAH is a rare but potentially fatal complication in adult IIM patients. Compared with the incidence of 8 %‒12 % in Ssc, the incidence of PAH in IIM patients of this cohort was 5.0 %, which was already higher than that in preceding study.^6^^,^^15^ Despite the comparably low incidence, the mortality rate of IIM patients with PAH was up to 48.0 % within six months. The high mortality rate made the early identification and timely intervention of PAH in IIM patients important.

The pathophysiological mechanisms of PAH include progressive pulmonary vascular resistance and remodeling secondary to release of cytokines and chemokines, dysfunction of immune cells, hypoxia, programmed cell death of endothelial cells and smooth muscle cells.^16^, ^17^, ^18^, ^19^ Inflammation and autoimmunity, in particular, are taken as key contributors to the initiation and progression of CTD-PAH.^6^ The major pathways responsible for the pathogenesis of CTD-PAH include but were not limited to nitric oxide, endothelin 1 and prostacyclin pathways.^15^ Under such circumstance it is not hard to understand IIM patients with higher MYOACT score showed higher tendency to develop PAH since MYOACT score, which reflects the disease activity of IIM patients, is correlated with the inflammation level in these patients.^14^^,^^20^

Bacterial infection, pulmonary bacterial infection in particular, was a common complication in IIM patients owing to the potent immunosuppressive medications as well as the immune dysregulation.^21^ In this study, bacterial infection (mostly pulmonary bacterial infection) was found to be significantly correlated with PAH in IIM patients. The correlation might be attributed to two aspects. On one hand, bacterial infection was comparably frequent in IIM patients and increased the inflammatory level in these patients.^22^ On the other hand, severe pulmonary bacterial infection led to hypoxia, which has already been proved to play a role in initiation and development of PAH.^23^^,^^24^ In preceding studies, PAH was also reported to be associated with infection of Human Immunodeficiency Virus (HIV), COVID-19, etc.^25^^,^^26^ The role and mechanism of infection in development of PAH demands further investigation.

Apart from the conventionally recommended PAH-specific targeted therapy, namely prostacyclin analogues and prostacyclin receptor agonists, endothelin receptor antagonists, as well as Phosphodiesterase-5 Inhibitors (PDE-5i) and guanylate cyclase stimulators, the role of immunosuppressive agents has also been emphasized in CTD-PAH.^6^ Potent immunosuppressive medications alleviate the inflammation and immune dysfunction in CTD patients and thus help treating CTD-PAH. As per the results of preceding studies, combined therapies of steroid and cyclophosphamide or mycophenolate may lead to clinical improvement in patients with PAH associated with Systemic Lupus Erythematosus (SLE) or Mixed Connective Tissue Disease (MCTD), but fail to show satisfying response in Ssc-PAH.^27^ In the present study, combined therapies of steroid, DMARDs, Intravenous Immunoglobulin (IVIG) or Janus Kinase (JAK) inhibitors did not show efficacy in reducing the incidence of PAH. However, the monotherapy of steroid, which was more commonly seen in IIM patients hospitalized at the department of respirology, infection or neurology, was found to be correlated with the development of PAH after multivariate logistic regression. Unlike the idiopathic form, inflammation and autoimmunity are believed to contribute to the initiation and progression of CTD-PAH via endothelin 1, nitric oxide, and prostacyclin pathways.^6^^,^^15^ Infiltrating macrophages and lymphocytes, complement, rheumatoid factors and antinuclear antibodies were detected in the pulmonary vessels of patients with CTD-PAH, which also indicated the role of autoimmunity in remodeling of pulmonary vasculature.^6^^,^^15^ Potent immunosuppressive regimens, combined therapies in particular, are thus essential in reducing the initiation and development of PAH in IIM patients. Additionally, only three out of seven moderate or severe PAH patients received endothelin receptor antagonists, all of them survived over 16-months. The limited proportion of IIM patients with PAH under PAH-targeted therapy reminded us of the ignorance of PAH-targeted medication in these patients. Due to the small sample size, however, no significant difference was observed in patients under endothelin receptor antagonists and those without.

Among different CTDs, Ssc has already been found to have the highest incidence of PAH. Preceding studies concerning CTD-PAH was largely focused on Ssc-PAH and identified various serum cytokines that might be related to development of PAH via different detecting techniques.^28^, ^29^, ^30^, ^31^ In this study, the authors revealed that IL-17A was correlated with complication of PAH in IIM patients. Previously IL-17A and IL-17A-producing cells have been identified to affect focal inflammation, structural damage and new bone formation in psoriatic arthritis and ankylosing spondylitis.^32^ Besides, IL-17 was also found to take part in initiation and development of experimental PAH via mice model, human pulmonary arterial endothelial cells or human pulmonary arterial smooth muscle cells.^33^, ^34^, ^35^ Targeting IL-17 would attenuate hypoxia-induced PAH through downregulation of β-catenin.^35^ To date, this is the first study digging into alterations of serum cytokines in IIM patients with PAH. The detailed mechanism behind IL-17A and CTD-PAH demand further exploration in the future.

Positivity of anti-SRP antibody is specific in the IMNM subtype of IIM, featuring severe limbic skeletal muscle involvement and frequent respiratory muscle involvement secondary to inflammatory infiltration, reticulum stress-induced autophagy pathway as well as necroptosis.^36^, ^37^, ^38^ Extramuscular involvement in SRP-IMNM mostly focused on interstitial lung disease, myocardial injury, etc.^37^ Previously few cases of SRP-IMNM patient reported complication of PAH.^39^ The clinical association between anti-SRP antibody and PAH has never been identified. In the present cohort, positivity of anti-SRP antibody was found to be significantly correlated with development of PAH in IIM patients. The detailed mechanism of PAH in SRP-IMNM patients remains an enigma. In preceding studies, complement membrane attack complex was found to participate in the muscular injury of IMNM. Besides, complement membrane attack complex was not only expressed in necrotic muscle fibers, but also positive in vascular endothelial cells and smooth muscle layer.^36^, ^37^, ^38^ Meanwhile the function of complement membrane attack complex has also been reported to contribute to PAH in Ssc.^40^ Whether complement membrane attack complex takes part in development of PAH in IIM, and the detailed upstream/downstream mechanisms, demand further clinical and laboratory research.

Recent years have seen aging as a research focus in different areas like cardiology, rheumatology and respirology. In preceding studies, PAH was found to be more common in older population of SSc, HIV, obstructive hypertrophic cardiomyopathy.^5^^,^^6^ Meanwhile aging was also found to be correlated with complication of PAH in this IIM cohort. A BAIMS model for prediction and early identification of PAH in IIM patients was constructed, with comparably satisfying predictive value through validation of decision tree analysis as well as ROC curve analysis. Preceding predictive models in IIM patients mostly focused on RP-ILD, secondary hemophagocytic lymphohistiocytosis and prognosis.^10^^,^^14^ To date, the BAIMS model is the first predictive model for PAH in adult IIM patients but still demands further verification in different IIM cohorts or subgroups.

Several limitations existed in this study. Due to the small sample of IIM patients with PAH in the cohort, validation of the BAIMS model was carried out in the original research cohort instead of a new validation cohort. To focus more on the clinical characteristics and peripheral serum biomarkers, cardiac imaging factors was not incorporated into this study. Although the patients in different divisions of FAHZJU reflect an overall circumstance in southeastern China, the cohort could only provide a regional (including multiple provinces), not a national insight into IIM and its complications.

## Conclusion

PAH is a scarce complication in adult IIM patients but frequently contributes to unfavorable outcome. Serum IL-17A and positivity of anti-SRP antibody, together with aging, disease activity, bacterial infection as well as steroid monotherapy, were significantly correlated with PAH in IIM patients. A BAIMS model was therefore established for prediction and early identification of PAH events and was initially proved efficient in different approaches of validations.

## Availability of data and materials

The source data was provided in Supplementary Table 6.

## Authors’ contributions

Study design: JLiang, XB, HC and JLin. Data collection: JLiang, XB, LW, YH, LX and HZ. Verification of IIM diagnosis: HC and YS. Identification of myocardial involvement: XC. Statistical analysis: JLiang. Writing: JLiang and XB. Proof reading: JLiang, XB, HC and JLin. All authors read and approved the final manuscript.

## Funding

This study was supported by the grants from 10.13039/501100001809National Natural Science Foundation of China (82201971) and 10.13039/501100004731Natural Science Foundation of Zhejiang Province (LQ22H100004).

## Declaration of competing interest

The authors declare no conflicts of interest.

## Appendix Supplementary materials

Image, application 1

Image, application 2
