# Supplementary material for: Risk factors and predictive model for pulmonary arterial hypertension in adult idiopathic-inflammatory-myopathy patients: A cross-sectional study
Source: Clinics (Sao Paulo). 2025 Mar 25;80:100621. doi: 10.1016/j.clinsp.2025.100621 (PMC11985143; doi:10.1016/j.clinsp.2025.100621)

**CLINICS-D-24-00973_Supplementary Material**

**Supplementary Table 1** Therapeutic regimens used in this cohort.

| Steroid monotherapy | Systemic Methylprednisolone (mPSL) or Prednisolone (PSL), the majority with a maximum dosage over 1 mg/kg/d (as calculated by prednisolone) |
| --- | --- |
| Combined therapy | Combined therapy of PSL/mPSL as well as Disease Modifying Anti-Rheumatic Drugs (DMARDs) or Janus Kinase (JAK) inhibitors, with or without intravenous immunoglobulin (IVIG). |
| The DMARDs applied to these patients encompassed Methotrexate, Cyclosporine, Tacrolimus, Mycophenolate, Thalidomide, Hydroxychloroquine and Cyclophosphamide. | |
| The JAK inhibitors used in this cohort were Baricitinib and Tofacitinib. | |

**Supplementary Table 2** Comparisons of multiple factors between PAH and non-PAH groups.

| **Factors** | **PAH (25)** | **Non-PAH (479)** | **p-value** |
| --- | --- | --- | --- |
| Age(y) | 70.00 (60.50, 78.00) | 57.00 (48.00, 65.00) | <0.001 |
| Sex(male/female) | 9/16 | 152/327 | 0.656 |
| Course of disease(m) | 6.00 (1.00, 33.00) | 3.00 (1.00, 12.00) | 0.158 |
| Duration of diagnosis delay (m) | 2.00 (0.50, 28.50) | 2.00 (0.75, 5.00) | 0.572 |
| **Clinical manifestations or complications** |  |  |  |
| Heliotrope rash | 9 (36.0%) | 175 (36.5%) | 0.957 |
| Gottron’s sign | 13 (52.0%) | 300 (62.6%) | 0.285 |
| Periungual erythema | 1 (4.0%) | 61 (12.7%) | 0.344 |
| Mechanic’s hand | 4 (16.0%) | 102 (21.3%) | 0.527 |
| Raynaud sign | 1 (4.0%) | 29 (6.1%) | 1.000 |
| Muscle pain | 12 (48.0%) | 236 (49.3%) | 0.902 |
| Muscle weakness | 19 (76.0%) | 295 (61.6%) | 0.147 |
| Joint pain | 1 (4.0%) | 112 (23.4%) | 0.023 |
| Joint swelling | 0 (0.0%) | 66 (13.8%) | 0.061 |
| Pharyngeal muscle involvement | 3 (12.0%) | 34 (7.1%) | 0.417 |
| Articulator muscle involvement | 0 (0.0%) | 17 (3.5%) | 1.000 |
| Respiratory muscle involvement | 1 (4.0%) | 8 (1.7%) | 0.370 |
| Lymphadenectasis | 14 (56.0%) | 197 (41.1%) | 0.142 |
| Hepatomegaly | 0 (0.0%) | 5 (1.0%) | 1.000 |
| Splenomegaly | 4 (16.0%) | 44 (9.2%) | 0.283 |
| Bacterial infection | 12 (48.0%) | 79 (16.5%) | <0.001 |
| Fungal infection | 4 (16.0%) | 51 (10.6%) | 0.338 |
| Tuberculosis infection | 1 (4.0%) | 10 (2.1%) | 0.432 |
| EBV infection | 7 (28.0%) | 63 (13.2%) | 0.066 |
| CMV infection | 1 (4.0%) | 18 (3.8%) | 1.000 |
| RP-ILD | 11 (44.0%) | 92 (19.2%) | 0.003 |
| Pulmonary hypertension | 1 (4.0%) | 2 (0.4%) | 0.142 |
| MI | 12 (48.0%) | 77 (16.1%) | <0.001 |
| Mediastinal emphysema | 2 (8.0%) | 12 (2.5%) | 0.149 |
| Gastrointestinal hemorrhage | 4 (16.0%) | 21 (4.4%) | 0.029 |
| Carcinoma | 1 (4.0%) | 57 (11.9%) | 0.340 |
| Smoking | 4 (16.0%) | 85 (17.7%) | 1.000 |
| Alcoholic abuse | 4 (16.0%) | 78 (16.3%) | 1.000 |
| Hypertension | 6 (24.0%) | 117 (24.4%) | 0.961 |
| Diabetes | 3 (12.0%) | 58 (12.1%) | 1.000 |
| Allergic history | 2 (8.0%) | 63 (13.2%) | 0.758 |
| **Disease activity** |  |  |  |
| MYOACT score | 12.00 (9.00, 15.00) | 8.00 (6.00, 12.00) | <0.001 |
| **Laboratory findings** |  |  |  |
| CD3^+^CD4^+^ lymphocytes (%) | 37.73±11.10 | 40.02±12.53 | 0.373 |
| CD3^+^CD8^+^ lymphocytes (%) | 33.67 (21.75, 40.24) | 23.90 (17.46, 32.20) | 0.012 |
| CD4^+^/ CD8^+^ Ratio | 1.15 (0.84, 2.09) | 1.66 (1.07, 2.53) | 0.043 |
| CD3^-^CD16^+^CD56^+^ lymphocytes (%) | 14.90 (5.62, 21.40) | 10.50 (6.20, 16.70) | 0.276 |
| CD3^-^CD19^+^ lymphocytes (%) | 6.50 (3.55, 19.47) | 16.80 (10.20, 23.20) | 0.001 |
| IL-2 (pg/ml) | 0.67 (0.10, 3.12) | 0.41 (0.10, 1.35) | 0.068 |
| IL-4 (pg/ml) | 0.93 (0.10, 3.00) | 0.47 (0.10, 1.59) | 0.094 |
| IL-6 (pg/ml) | 11.84 (5.21, 45.68) | 6.41 (3.21, 19.20) | 0.070 |
| IL-10 (pg/ml) | 4.04 (1.81, 5.31) | 2.74 (1.42, 4.48) | 0.068 |
| TNF-α (pg/ml) | 2.45 (0.10, 6.87) | 1.29 (0.10, 3.23) | 0.192 |
| IFN-γ (pg/ml) | 2.51 (0.10, 7.90) | 0.95 (0.10, 4.53) | 0.165 |
| IL-17A (pg/ml) | 0.53 (0.10, 24.23) | 0.10 (0.10, 5.22) | 0.038 |
| CRP (mg/L) | 22.50 (4.85, 74.90) | 4.70 (3.00, 18.89) | 0.004 |
| ESR (mm/h) | 23.00 (7.00, 63.00) | 16.00 (7.00, 33.00) | 0.284 |
| Ferritin (ng/mL) | 531.30 (206.40, 1106.35) | 335.20 (139.20, 871.30) | 0.236 |
| Alpha-fetoprotein (ng/mL) | 1.80 (1.25, 2.75) | 2.30 (1.66, 3.40) | 0.055 |
| Carcinoembryonic antigen (ng/mL) | 3.50 (1.45, 6.35) | 2.50 (1.42, 4.30) | 0.409 |
| CA125 (U/mL) | 46.20 (24.75, 86.10) | 16.50 (10.70, 27.50) | <0.001 |
| CA199 (U/mL) | 9.00 (4.10, 24.50) | 5.60 (2.80, 13.90) | 0.081 |
| LDH (U/L) | 364.00 (276.50, 720.50) | 302.00 (229.00, 455.00) | 0.051 |
| CK (U/L) | 480.00 (103.50, 2338.50) | 166.00 (52.00, 829.00) | 0.024 |
| CK-MB (U/L) | 24.00 (16.00,61.00) | 21.00 (15.00, 35.00) | 0.222 |
| **Myositis-specific antibodies & Myositis-associated antibodies** |  |  |  |
| Anti-MDA5 | 3 (12.0%) | 98 (20.5%) | 0.303 |
| Anti-PL-7 | 3 (12.0%) | 50 (10.4%) | 0.738 |
| Anti-PL-12 | 1 (4.0%) | 28 ((5.8%) | 1.000 |
| Anti-EJ | 4 (16.0%) | 30 (6.3%) | 0.079 |
| Anti-OJ | 1 (4.0%) | 10 (2.1%) | 0.432 |
| Anti-Jo-1 | 4 (16.0%) | 90 (18.8%) | 1.000 |
| Anti-TIF1γ | 2 (8.0%) | 36 (7.5%) | 1.000 |
| Anti-Mi-2α | 0 (0.0%) | 20 (4.2%) | 0.615 |
| Anti-Mi-2β | 1 (4.0%) | 24 (5.0%) | 1.000 |
| Anti-SAE1 | 1 (4.0%) | 8 (1.7%) | 0.370 |
| Anti-NXP2 | 0 (0.0%) | 25 (5.2%) | 0.627 |
| Anti-SRP | 4 (16.0%) | 22 (4.6%) | 0.034 |
| HMGCR | 0 (0.0%) | 9 (1.9%) | 1.000 |
| Anti-Ku | 1 (4.0%) | 14 (2.9%) | 0.539 |
| Anti-PM-Scl75 | 0 (0.0%) | 18 (3.8%) | 1.000 |
| Anti-PM-Scl100 | 1 (4.0%) | 7 (1.5%) | 0.336 |
| Anti-Ro-52 | 13 (52.0%) | 260 (54.3%) | 0.824 |
| KS | 0 (0.0%) | 1 (0.2%) | 1.000 |
| Hatyr | 1 (4.0%) | 0 (0.0%) | 0.050 |
| cN1A | 0 (0.0%) | 2 (0.4%) | 1.000 |
| **Therapies** |  |  |  |
| Steroid monotherapy | 12 (48.0%) | 88 (18.4%) | 0.001 |
| Maximum dosage of steroid | 100.00 (62.50, 150.00) | 75.00 (50.00, 100.00) | 0.228 |
| Steroid+DMARDs | 6 (24.0%) | 271 (56.6%) | 0.001 |
| [Steroid+IVIG](http://www.baidu.com/link?url=_srwKTXKnet8GknUvvs0xyTJdpfNOQtIDWHWhe_U5wypEldT9OPh2gCg3LsSDR-5CpyLTLOBAy4p4ov8wle8F6_YWPs4sPX-lyXINgDKaDW) | 5 (20.0%) | 41 (8.6%) | 0.067 |
| [Steroid+DMARDs+IVIG](http://www.baidu.com/link?url=uciYHxddnq2QF5VJVWJRCy7Q7nEAXlzzmiKvgGzZkrPg72XHW0qrc1acnFRmU-CtSPSZqd_rW-WBKuZFe0OpuS_h9gOsjyItDqvwfb_UtbdGjXJvU0FWCCPVF1qaXYLk) | 1 (4.0%) | 52 (10.9%) | 0.500 |
| Steroid+JAK inhibitor | 1 (4.0%) | 10 (2.1%) | 0.432 |
| Steroid+DMARDs+JAK inhibitor | 0 (0.0%) | 10 (2.1%) | 1.000 |
| Steroid+IVIG+JAK inhibitor | 0 (0.0%) | 3 (0.6%) | 1.000 |
| Steroid+DMARDs+IVIG+JAK inhibitor | 0 (0.0%) | 2 (0.4%) | 1.000 |
| **IIM subtypes** |  |  |  |
| DM | 13 (52.0%) | 295 (61.6%) | 0.338 |
| PM | 6 (24.0%) | 93 (19.4%) | 0.605 |
| ADM | 2 (8.0%) | 56 (11.7%) | 0.756 |
| IMNM | 4 (16.0%) | 33 (6.9%) | 0.102 |
| IBM | 0 (0.0%) | 2 (0.4%) | 1.000 |

PAH, Pulmonary Artery Hypertension; P-adjusted, Adjusted p-value after false discovery rate correction; y, years; m, months; EBV, Epstein-Barr virus; CMV, Cytomegalovirus; RP-ILD, Rapidly Progressive Interstitial Lung Disease; MI, Myocardial Involvement; MYOACT, Myositis Disease Activity Assessment Visual Analogue Scales; CD, Clusters of differentiation; IL, Interleukin; TNF, Tumor Necrosis Factor; IFN, Interferon; CRP, C-Reactive Protein; ESR, Erythrocyte Sedimentation Rate; LDH, Lactate Dehydrogenase; CK, Creatine Kinase; CK-MB, Creatine Kinase Isoenzymes; DMARDs, Disease-Modifying Anti-Rheumatic Drugs; IVIG, Intravenous Immunoglobulin; JAK, Janus Kinase; IIM, Idiopathic Inflammatory Myopathy; DM, Dermatomyositis; PM, Polymyositis; ADM, Amyopathic Dermatomyositis; IMNM, Immune-related Necrotizing Myopathy; IBM, Inclusion Body Myositis.

**Supplementary Table 3** Univariate logistic regression analyses of PAH among IIM patients.

| **Factors** | **p-value** | **OR value** | **95% Confidence interval** |
| --- | --- | --- | --- |
| Age (y) | <0.001 | 1.096 | 1.053~1.140 |
| Sex (male/female) | 0.656 | 1.210 | 0.523~2.800 |
| Course of disease (m) | 0.311 | 1.003 | 0.997~1.010 |
| Duration of diagnosis delay (m) | 0.155 | 1.010 | 0.996~1.025 |
| **Clinical manifestations or complications** |  |  |  |
| Heliotrope rash | 0.957 | 0.977 | 0.423~2.258 |
| Gottron’s sign | 0.289 | 0.646 | 0.289~1.447 |
| Periungual erythema | 0.224 | 0.286 | 0.038~2.149 |
| Mechanic’s hand | 0.529 | 0.704 | 0.236~2.097 |
| Raynaud sign | 0.675 | 0.647 | 0.084~4.949 |
| Muscle pain | 0.902 | 0.950 | 0.425~2.125 |
| Muscle weakness | 0.154 | 1.975 | 0.775~5.037 |
| Joint pain | 0.052 | 0.137 | 0.018~1.021 |
| Joint swelling | 0.997 | <0.001 | <0.001 ~ >1000.000 |
| Pharyngeal muscle involvement | 0.366 | 1.785 | 0.508 ~ 6.265 |
| Articulator muscle involvement | 0.999 | <0.001 | <0.001 ~ >1000.000 |
| Respiratory muscle involvement | 0.407 | 2.453 | 0.295 ~ 20.415 |
| Lymphadenectasis | 0.147 | 1.822 | 0.810 ~ 4.097 |
| Hepatomegaly | 0.999 | <0.001 | <0.001 ~ >1000.000 |
| Splenomegaly | 0.265 | 1.883 | 0.619 ~ 5.733 |
| Bacterial infection | <0.001 | 4.674 | 2.057 ~ 10.621 |
| Fungal infection | 0.407 | 1.599 | 0.528 ~ 4.841 |
| Tuberculosis infection | 0.531 | 1.954 | 0.240 ~ 15.897 |
| EBV infection | 0.043 | 2.568 | 1.031 ~ 6.395 |
| CMV infection | 0.951 | 1.067 | 0.137 ~ 8.331 |
| RP-ILD | 0.004 | 3.305 | 1.453 ~ 7.518 |
| Pulmonary embolism | 0.065 | 0.937 | 0.870 ~ 113.470 |
| MI | <0.001 | 4.819 | 2.119 ~ 10.960 |
| Mediastinal emphysema | 0.124 | 3.384 | 0.715 ~ 16.014 |
| Gastrointestinal hemorrhage | 0.016 | 4.154 | 1.308 ~ 13.189 |
| Carcinoma | 0.254 | 0.308 | 0.041 ~ 2.324 |
| Smoking | 0.824 | 0.883 | 0.295 ~ 2.638 |
| Alcoholic abuse | 0.970 | 0.979 | 0.327 ~ 2.931 |
| Hypertension | 0.961 | 0.977 | 0.381 ~ 2.504 |
| Diabetes | 0.987 | 0.990 | 0.287 ~ 3.411 |
| Allergic history | 0.459 | 0.574 | 0.132 ~ 2.495 |
| **Disease activity** |  |  |  |
| MYOACT score | <0.001 | 1.232 | 1.110 ~ 1.369 |
| **Laboratory findings** |  |  |  |
| CD3^+^CD4^+^ lymphocytes (%) | 0.373 | 0.985 | 0.954 ~ 1.018 |
| CD3^+^CD8^+^ lymphocytes (%) | 0.033 | 1.034 | 1.003 ~ 1.066 |
| CD4^+^/ CD8^+^ Ratio | 0.067 | 0.665 | 0.430 ~ 1.028 |
| CD3^-^CD16^+^CD56^+^ lymphocytes (%) | 0.168 | 1.024 | 0.990 ~ 1.058 |
| CD3^-^CD19^+^ lymphocytes (%) | 0.003 | 0.921 | 0.872 ~ 0.973 |
| IL-2 (pg/mL) | 0.107 | 1.048 | 0.990 ~ 1.109 |
| IL-4 (pg/mL) | 0.152 | 1.094 | 0.967 ~ 1.238 |
| IL-6 (pg/mL) | 0.023 | 1.001 | 1.000 ~ 1.002 |
| IL-10 (pg/mL) | 0.863 | 1.000 | 0.994 ~ 1.005 |
| TNF-α (pg/mL) | 0.767 | 1.006 | 0.969 ~ 1.044 |
| IFN-γ (pg/mL) | 0.069 | 1.011 | 0.999 ~ 1.022 |
| IL-17A (pg/mL) | 0.033 | 1.016 | 1.001 ~ 1.032 |
| CRP (mg/L) | 0.001 | 1.010 | 1.004 ~ 1.016 |
| ESR (mm/h) | 0.025 | 1.016 | 1.002 ~ 1.031 |
| Ferritin (ng/mL) | 0.020 | 1.000 | 1.000 ~ 1.000 |
| Alpha-fetoprotein (ng/mL) | 0.101 | 0.738 | 0.513 ~ 1.061 |
| Carcinoembryonic antigen (ng/mL) | 0.909 | 1.002 | 0.969 ~ 1.036 |
| CA125 (U/mL) | 0.179 | 1.001 | 0.999 ~ 1.003 |
| CA199 (U/L | 0.646 | 1.001 | 0.997 ~ 1.004 |
| LDH (U/L) | 0.529 | 1.000 | 1.000 ~ 1.001 |
| CK (U/L) | 0.792 | 1.000 | 1.000 ~ 1.000 |
| CK-MB (U/L) | 0.879 | 1.000 | 0.997 ~ 1.003 |
| **Myositis-specific antibodies & Myositis-associated antibodies** |  |  |  |
| Anti-MDA5 | 0.311 | 0.530 | 0.155 ~ 1.807 |
| Anti-PL-7 | 0.804 | 1.170 | 0.338 ~ 4.048 |
| Anti-PL-12 | 0.701 | 0.671 | 0.088 ~ 5.143 |
| Anti-EJ | 0.070 | 2.851 | 0.920 ~ 8.837 |
| Anti-OJ | 0.531 | 1.954 | 0.240 ~ 15.897 |
| Anti-Jo-1 | 0.727 | 0.823 | 0.276 ~ 2.457 |
| Anti-TIF1γ | 0.929 | 1.070 | 0.243 ~ 4.721 |
| Anti-Mi-2α | 0.998 | <0.001 | <0.001 ~ >1000.000 |
| Anti-Mi-2β | 0.821 | 0.790 | 0.103 ~ 6.088 |
| Anti-SAE1 | 0.407 | 2.453 | 0.295 ~ 20.415 |
| Anti-NXP2 | 0.998 | <0.001 | <0.001 ~ >1000.000 |
| Anti-SRP | 0.019 | 3.957 | 1.251 ~ 12.517 |
| HMGCR | 0.999 | <0.001 | <0.001 ~ >1000.000 |
| Anti-Ku | 0.758 | 1.384 | 0.175 ~ 10.965 |
| Anti-PM-Scl75 | 0.998 | <0.001 | <0.001 ~ >1000.000 |
| Anti-PM-Scl100 | 0.343 | 2.810 | 0.332 ~ 23.761 |
| Anti-Ro-52 | 0.824 | 0.913 | 0.408 ~ 2.041 |
| KS | 1.000 | <0.001 | <0.001 ~ >1000.000 |
| Hatyr | 1.000 | >1000.000 | <0.001 ~ >1000.000 |
| cN1A | 0.999 | <0.001 | <0.001 ~ >1000.000 |
| **Therapies** |  |  |  |
| Steroid monotherapy | 0.001 | 4.101 | 1.810 ~ 9.293 |
| Maximum dosage of steroid | 0.759 | 1.000 | 0.998 ~ 1.003 |
| Steroid+DMARDs | 0.003 | 0.242 | 0.095 ~ 0.618 |
| [Steroid+IVIG](http://www.baidu.com/link?url=_srwKTXKnet8GknUvvs0xyTJdpfNOQtIDWHWhe_U5wypEldT9OPh2gCg3LsSDR-5CpyLTLOBAy4p4ov8wle8F6_YWPs4sPX-lyXINgDKaDW) | 0.062 | 2.671 | 0.953 ~ 7.488 |
| [Steroid+DMARDs+IVIG](http://www.baidu.com/link?url=uciYHxddnq2QF5VJVWJRCy7Q7nEAXlzzmiKvgGzZkrPg72XHW0qrc1acnFRmU-CtSPSZqd_rW-WBKuZFe0OpuS_h9gOsjyItDqvwfb_UtbdGjXJvU0FWCCPVF1qaXYLk) | 0.298 | 0.342 | 0.045 ~ 2.582 |
| Steroid+JAK inhibitor | 0.531 | 1.954 | 0.240 ~ 15.897 |
| Steroid+DMARDs+JAK inhibitor | 0.999 | <0.001 | <0.001 ~ >1000.000 |
| Steroid+IVIG+JAK inhibitor | 0.999 | <0.001 | <0.001 ~ >1000.000 |
| Steroid+DMARDs+IVIG+JAK inhibitor | 0.999 | <0.001 | <0.001 ~ >1000.000 |
| **IIM subtypes** |  |  |  |
| DM | 0.340 | 0.676 | 0.302 ~ 1.513 |
| PM | 0.575 | 1.311 | 0.509 ~ 3.373 |
| ADM | 0.576 | 0.657 | 0.151 ~ 2.861 |
| IMNM | 0.100 | 2.574 | 0.835 ~ 7.939 |
| IBM | 0.999 | <0.001 | <0.001 ~ >1000.000 |

PAH, Pulmonary Artery Hypertension; OR, Odds Ratio; P-adjusted, Adjusted p-value after false discovery rate correction; y, years; m, months; EBV, Epstein-Barr virus; CMV, Cytomegalovirus; RP-ILD, Rapidly Progressive Interstitial Lung Disease; MI, Myocardial Involvement; MYOACT, Myositis Disease Activity Assessment Visual Analogue Scales; CD, Clusters of Differentiation; IL, Interleukin; TNF, Tumor Necrosis Factor; IFN, Interferon; CRP, C-Reactive Protein; ESR, Erythrocyte Sedimentation Rate; LDH, Lactate Dehydrogenase; CK, Creatine Kinase; CK-MB, Creatine Kinase Isoenzymes; DMARDs, Disease-Modifying Anti-Rheumatic Drugs; IVIG, Intravenous Immunoglobulin; JAK, Janus Kinase; IIM, Idiopathic Inflammatory Myopathy; DM, Dermatomyositis; PM, Polymyositis; ADM, Amyopathic Dermatomyositis; IMNM, Immune-related Necrotizing Myopathy; IBM, Inclusion Body Myositis.

**Supplementary Table 4** BAIMS score for prediction of PAH in IIM.

| **Factors** | **Value** | **Score^a^** |
| --- | --- | --- |
| Age (y) | >65 | 1 |
| Bacterial infeciton | Positive | 1 |
| MYOACT score | >8 | 1 |
| IL-17A (pg/mL) | >0.15 | 1 |
| SRP | Positive | 1 |
| Steroid montherapy | Positive | 1 |
| Cumulative score | / | 0 ~ 6 |

PAH, Pulmonary Artery Hypertension; IIM, Idiopathic Inflammatory Myopathy; y, years; MYOACT, Myositis Disease Activity Assessment Visual Analogue Scales; IL, Interleukin.

^a^ 0 or 1 for each item.

**Supplementary Table 5** Distribution of PAH and non-PAH in IIM patients with different BAIMS score.

| **BAIMS score** | **Total patients (n)** | **PAH % (n)** | **Non-PAH % (n)** |
| --- | --- | --- | --- |
| 0 ~ 1 | 252 | 0.4% (1) | 99.6% (251) |
| 2 ~ 3 | 221 | 5.9% (13) | 94.1% (208) |
| 4 ~ 5 | 31 | 35.5% (11) | 64.5% (20) |

*PAH, Pulmonary Artery Hypertension; IIM, Idiopathic Inflammatory Myopathy.

**Supplementary Table 6** Source data for this manuscript.

The source data will be provided in an excel file.

**Supplementary Data 1** Detailed protocols for detection of peripheral lymphocyte subsets, serum cytokines as well as myositis specific/associated antibodies.

| Peripheral lymphocyte subsets were assessed and quantified as percentages of CD3^+^CD4^+^, CD3^+^CD8^+^, CD3^-^CD16^+^CD56^+^ and CD3^-^ CD19^+^ cells utilizing CD45-PE, CD3-PC5, CD4-FITC, CD8-PE, CD3-FITC-CD (16+56)-PE and CD19-FITC mouse anti-human fluorescence monoclonal antibodies (BD Bioscience) as well as the BD FACScanto™ II flow cytometer (Becton Dickinson, San Jose, CA, USA). At the same time serum levels of Tumor Necrosis Factor (TNF)-α, Interferon (IFN)-γ, Interleulin-2 (IL-2), IL-4, IL-6, IL-10 and IL-17A, were determined with the Cytometric Bead Array (CBA) kit BD™ CBA Human Th1/Th2/TH17 Cytokine Kit (BD Biosciences, San Jose, CA, USA) as well as the flow cytometer (described above). Evaluation of different cytokine levels was carried out as per the manufacturer’s instructions. The lower and upper limits of cytokine assessment were 0.10 pg/mL and 5000.00 pg/mL, respectively. The produced data were engendered in graphical and tabular format utilizing FCAP Array™ software (BD Biosciences, San Jose, CA, USA). The MSAs (anti-MDA5, anti-Jo-1, anti-PL-7, anti-PL-12, anti-KS, anti-Hatyr, anti-EJ, anti-OJ, anti-TIF1γ, anti-NXP2, anti-SAE1, anti-Mi-2α, anti-Mi-2β, anti-SRP, anti-HMGCR and anti-cN1A) and MAAs (anti-PM-Scl75, anti-PM-Scl100, anti-Ro-52 and anti-Ku) were measured by an immunoblotting assay (EUROLINE Myositis Research Profile, EUROIMMUN, Lübeck, Germany)and the ELISA (INOVA, Falls Church, Virginia, United States) as per the manufacturers’ protocols. |
| --- |

**Supplementary Figure 1** **Enrollment and groupings of this IIM cohort.** IIM, Idiopathic Inflammatory Myopathy; PAH, Pulmonary Arterial Hypertension.


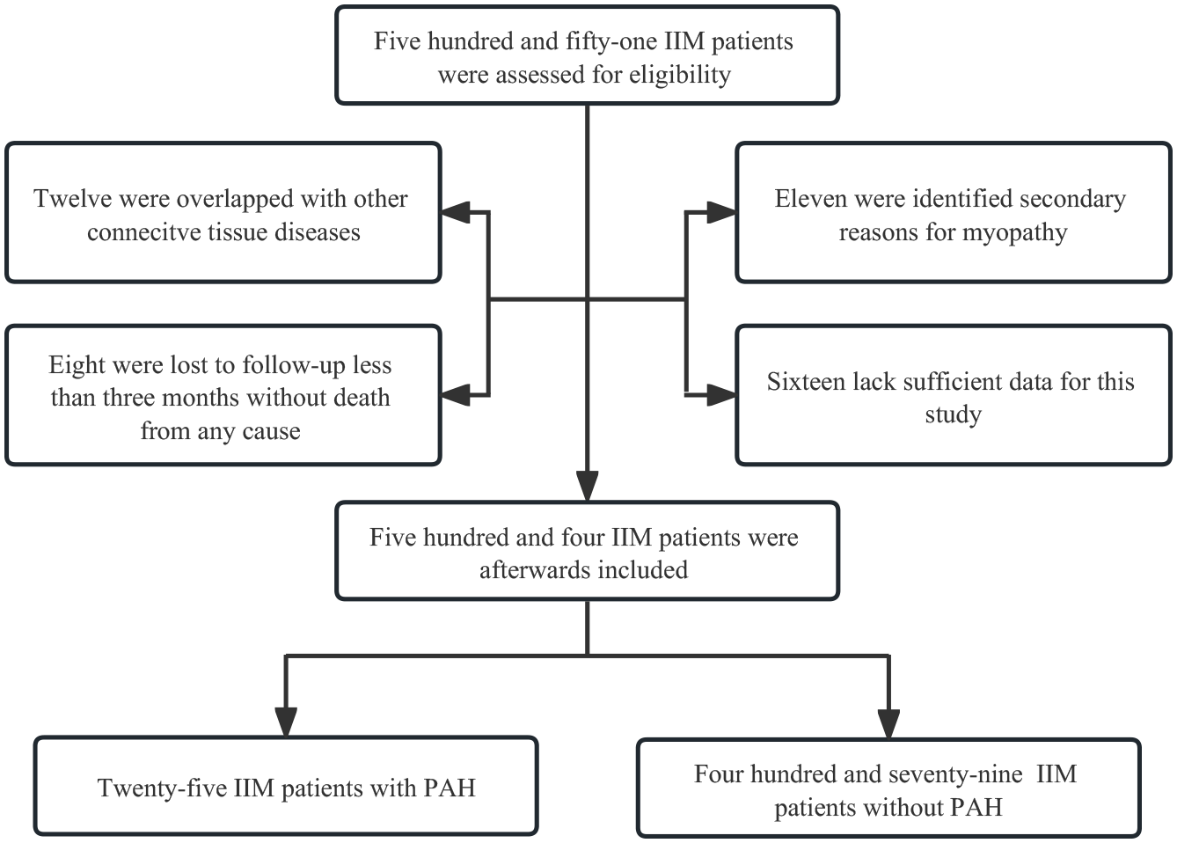


**Supplementary Figure 2 ROC curve of age, MYOACT score and IL-17A predicting PAH.** (A) ROC curve of age predicting PAH. (B) ROC curve of MYOACT score predicting PAH. (C) ROC curve of IL-17A predicting PAH. ROC, Receiver Operating Characteristic; MYOACT, Myositis Disease Activity Assessment Visual Analogue Scales; IL, Interleukin; PAH, Pulmonary Arterial Hypertension; AUC, Area Under the Curve.


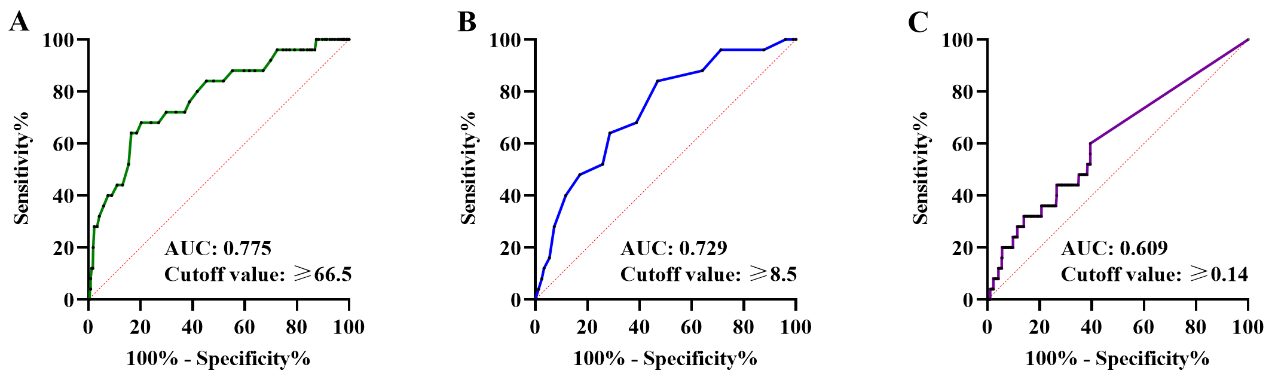

Supplement: Supplementary file 1 [file mmc1.docx]
